# Efficient clinical evaluation of guideline quality: development and testing of a new tool

**DOI:** 10.1186/1471-2288-14-63

**Published:** 2014-05-10

**Authors:** Karen Grimmer, Janine Margarita Dizon, Steve Milanese, Ellena King, Kate Beaton, Olivia Thorpe, Lucylynn Lizarondo, Julie Luker, Zuzana Machotka, Saravana Kumar

**Affiliations:** 1 International Centre for Allied Health Evidence (iCAHE), University of South Australia, City East Campus, School of Health Sciences, Centenary, GPO box 2471, Adelaide 5001, Australia; 2 College of Rehabilitation Sciences, University of Santo Tomas, St. Martin de Porres Building University of Santo Tomas Espana, Manila 1083, Philippines; 3 Florey Institute of Neurosciences & Mental Health, University of Melbourne, NHMRC Research Fellow, Melbourne, Victoria, Australia

**Keywords:** Guideline quality assessment, Psychometric testing, AGREE II instrument, iCAHE guideline quality checklist

## Abstract

**Background:**

Evaluating the methodological quality of clinical practice guidelines is essential before deciding which ones which could best inform policy or practice. One current method of evaluating clinical guideline quality is the research-focused AGREE II instrument. This uses 23 questions scored 1–7, arranged in six domains, which requires at least two independent testers, and uses a formulaic weighted domain scoring system. Following feedback from time-poor clinicians, policy-makers and managers that this instrument did not suit clinical need, we developed and tested a simpler, shorter, binary scored instrument (the iCAHE Guideline Quality Checklist) designed for single users.

**Methods:**

Content and construct validity, inter-tester reliability and clinical utility were tested by comparing the new iCAHE Guideline Quality Checklist with the AGREE II instrument. Firstly the questions and domains in both instruments were compared. Six randomly-selected guidelines on a similar theme were then assessed by three independent testers with different experience in guideline quality assessment, using both instruments. Per guideline, weighted domain and total AGREE II scores were calculated, using the scoring rubric for three testers. Total iCAHE scores were calculated per guideline, per tester. The linear relationship between iCAHE and AGREE II scores was assessed using Pearson r correlation coefficients. Score differences between testers were assessed for the iCAHE Guideline Quality Checklist.

**Results:**

There were congruent questions in each instrument in four domains (Scope & Purpose, Stakeholder involvement, Underlying evidence/Rigour, Clarity). The iCAHE and AGREE II scores were moderate to strongly correlated for the six guidelines. There was generally good agreement between testers for iCAHE scores, irrespective of their experience. The iCAHE instrument was preferred by all testers, and took significantly less time to administer than the AGREE II instrument. However, the use of only three testers and six guidelines compromised study power, rendering this research as pilot investigations of the psychometric properties of the iCAHE instrument.

**Conclusion:**

The iCAHE Guideline Quality Checklist has promising psychometric properties and clinical utility.

## Background

An internet search will generally identify at least one clinical practice guideline for most common health questions. There is however, no standard approach to constructing clinical practice guidelines [1], despite clear developmental standards being established by internationally-respected groups. International organisations such as the Institute of Medicine [2] (IOM), World Health Organisation [3] (WHO), National Institute for Health and Clinical Excellence [4] (NICE) and Scottish Intercollegiate Guideline Network [5] (SIGN) all provide readily available resources to aid developers in producing high quality evidence based guidelines. However despite these valuable resources, variable quality clinical guidelines continue to be developed by many organisations in different countries, as the demand for evidence-based practice aids grows globally. For instance in Australia, more than 100 organisations are currently listed on the National Health and Medical Research Council (NHMRC) Clinical Practice Guidelines Portal as having an interest in guideline development [6]. A similar situation would be expected in most developed countries.

However, no matter how well constructed a clinical guideline is, access to a high quality evidence source alone does not necessarily result in translating that evidence into clinical practice. There is a large body of literature regarding barriers to evidence implementation particularly by clinicians. Barriers for clinician end-users can be grouped into individual beliefs, behaviours, disincentives to change, organisational and structural supports, education and cultural beliefs [7-9]. Globally, and across health disciplines, time, knowledge about the elements of quality guidelines, demonstrable incentives and accessibility to information on guidelines are universal barriers to implementing evidence in clinical guidelines into practice [10-13].

Thus clinicians wishing to identify the best quality clinical practice guidelines that could assist their clinical decisions, are faced with many difficult questions, such as ‘where to go for information’ , ‘which guideline to choose from the many of variable appearance and credibility’ , ‘how to efficiently choose a good guideline’ , and ‘how to determine its methodological rigour, relevance to clinical settings and applicability to clinical questions’. Lack of simple and efficient solutions to these questions may undermine commitment by clinicians, policy-makers and managers to putting best current evidence into practice.

There is no standard approach to assessing clinical practice guideline quality, which addresses the needs of researchers, methodologists, educators, clinicians, policy-makers and managers. In the literature, the most commonly-reported guideline quality instrument is the AGREE instrument [14,15]. This was developed primarily for guideline developers and researchers, to outline and measure core elements of guideline construction and implementation. The AGREE instrument (initially AGREE I [14], now AGREE II [15]) consists of six domains of ‘scope and purpose’ , ‘stakeholder involvement’ , ‘rigour of development’ , ‘clarity of presentation’ , ‘applicability’ , ‘editorial independence’. The AGREE II instrument [15] contains 23 questions in these domains, with each question scored using a 1–7 scale of perceived compliance. To calculate AGREE II instrument scores requires two or more testers (i.e., AGREE cannot be completed by only one tester), and a scoring rubric is provided to weight domain scores, depending on how many testers participate [15]. The reliability of the AGREE II instrument has been variably reported [16,17]. Recently a Guidelines International Network (GIN) panel [1] suggested that there were different quality domains (composition, decision-making process, conflicts of interest, guideline objective, development methods, evidence review, basis of recommendations, ratings of evidence and recommendations, guideline review, updating processes, and funding). However to date, no scoring system has been proposed for the GIN approach.

Our team from the International Centre for Allied Health Evidence (*i*CAHE) (University of South Australia, Australia) commenced a Guidelines Clearinghouse initiative on its website [18] in 2008, by collating clinical guidelines which address conditions of interest to iCAHE members (namely clinicians, managers and policy makers). While the primary end-users of the Guideline Clearing House were allied health clinicians, policy-makers and managers (in line with the iCAHE mission and funding imperatives), the Guideline Clearing House is also accessed by other health disciplines, as well as educators and researchers. The aim underpinning the Guidelines Clearing House initiative was to remove as many barriers as possible for website end-users regarding access to, and uptake of, good quality evidence-based information. This meant that not only should we make it as simple as possible for users to access a wide range of clinical guidelines, but also to provide an indication of methodological quality. We also recognised that we should provide a way for our website end-users to score the methodological quality of other guidelines that they might find through their own efforts.

During the development of the iCAHE Guideline Clearing House, discussions held with Australian policy makers, educators, clinicians and administrators identified the need for a psychometrically sound, efficient, simply scored quality assessment instrument that addressed important guideline quality criteria, and assisted end-users to make decisions on their own, in minutes, regarding clinical guideline quality. Consistent feedback was that the AGREE instrument was not appropriate in busy clinical settings, due to its number of questions, the complexity of the 1–7 scoring system, and the requirement for multiple testers to make a judgment on guideline quality. Moreover, our discussions highlighted that a clinically-oriented guideline quality assessment instrument should not concurrently consider relevance or applicability of recommendations to local clinical practice contexts. In clinical and policy settings it was essential to separate these issues. Once a guideline of good quality had been established, discussions could then occur regarding contextualisation to local practice settings, and then implementation.

This paper describes the development and psychometric testing of a simple, single-user clinical guideline methodological quality checklist designed for busy clinical and policy settings.

## Methods

### Ethics

Ethical approval was provided by the Human Research Ethics Committee, University of South Australia as part of a larger evidence-implementation project (P208/09).

### Developmental work

Elements of clinical guideline quality which were considered to be important by clinicians, managers and policy-makers were identified during the construction of the iCAHE Clinical Guideline Clearinghouse. Moreover, common methodological quality elements were identified from material developed by internationally-recognised guideline developers [2-6]. A draft 14 item instrument was constructed using binary assessment for each item (Yes, there was clear evidence that an item had been addressed, or No, there was not clear evidence that an item had been addressed). This is the same scoring approach as used in the PEDro critical appraisal instrument for randomised controlled trials [19], which would be familiar to many end-users of the iCAHE website via its Critical Appraisal Tools page [18]. The draft clinical guideline critical appraisal instrument was made available for public comment on the iCAHE website [18] in 2009 for three months. No changes to its format were suggested by 32 respondents, and the resultant iCAHE Guideline Quality Checklist has been in use since then, in its original form (see Additional file 1).

### Research questions

The following questions were asked during psychometric testing of the iCAHE Guideline Quality Checklist:

1. What are the psychometric properties (content and construct validity, and inter-tester reliability) of the iCAHE Guideline Quality Checklist, when compared with the AGREE II instrument [15]?

2. Can an inexperienced tester use the iCAHE Guideline Quality Checklist effectively?

3. Does the iCAHE Guideline Quality Checklist have similar clinical utility to the AGREE II instrument?

### Guideline assessment instrument scoring

Choosing the AGREE II instrument for validation purposes imposed constraints on what comparative testing could be undertaken. The AGREE II scoring rubric requires two or more testers, to produce tester-weighted scores for each of six domains. These scores provide no measure of variability, and individual AGREE II scores are not available. Thus tester differences in domain scores cannot be calculated. Moreover, the developers recommended that a total AGREE II score should not be calculated [15]. Conversely, the iCAHE instrument is designed to be scored by one tester, thus multiple tester data could be described by central tendency (average), Standard Deviations could be calculated and tests for homogeneity undertaken to assess tester differences.

### Psychometric testing

Content validity was assessed by aligning the questions in the iCAHE Guideline Quality Checklist with those in the AGREE II instrument, and identifying which of the AGREE II domains were assessed by the iCAHE instrument.

Construct validity was tested by the correlation between guideline quality scores from the iCAHE instrument and the AGREE II instrument. For this purpose, six clinical guidelines related to the management of traumatic brain injury were assessed. These guidelines were randomly selected from 53 systematically-identified guidelines collated for a large quality and safety project. Pearson correlation coefficients and 95% Confidence Intervals were applied to determine the strength of correlation between overall scores for iCAHE and AGREE II instruments, as well as individual testers’ scores using the iCAHE instrument.

Tester experience was assessed by employing three purposively-sampled testers with different experiences in guideline quality assessment. Each tester assessed the methodological quality of each guideline independently, using both the AGREE II and iCAHE checklists. Testers determined their own order of assessing guidelines, and with which tools. Tester 1 was an experienced guideline writer, and a developer of the iCAHE checklist; Tester 2 had moderate experience in guideline writing, and had some experience of using the iCAHE checklist, but no involvement with its development; and Tester 3 was a novice guideline assessor with no experience in using either instrument. No training was provided on how to use either instrument, and scores were not discussed.

Inter-rater reliability was determined by assessing differences between testers’ scores on the iCAHE instrument. Tester differences in scoring the AGREE II instrument domains could only be considered using the testers’ raw scores for each question (which is not recommended practice).

### Utility

The raters recorded the time spent scoring each guideline with each instrument. A semi-structured exit interview was conducted by an independent researcher with each individual rater, to identify their perspectives on the simplicity of scoring using each instrument, their preferences, and what underpinned these.

### Data management

iCAHE instrument scores were not reported per domain (as is required for the AGREE II instrument), as the intention of this instrument was to provide an efficient, global quality rating process per guideline. Thus, for the iCAHE Guideline Quality Checklist, percentage total quality scores were calculated per guideline per tester by converting ‘yes’ and ‘no’ responses to 1 or 0 respectively, then summing the total number of Yes scores, dividing this by the maximum possible score (14) and expressing this as a percentage.

The AGREE II checklist comprises six domains, each containing between 2 and 8 questions. Each question is scored with 7-point scale. Domain scores are calculated using the AGREE II guideline scoring rubric (agree@mcmaster.ca) [15]:

Obtained score – Minimum possible score

Maximum possible score – Minimum possible score

The variability of decision-making is not captured in the AGREE II rubric (i.e., a single value is reported with no information on range, or differences between testers). Although it is not recommended [15], we calculated a total AGREE II score for the purpose of this paper, by applying the same scoring rubric as above to all 23 questions, and expressing this as percentage of the possible total agreement score. This standardised comparison with the iCAHE Guideline Quality Checklist percentage-of-total scores. We also considered the raw tester scores for each AGREE II question to assess tester experience in determining AGREE II score distributions.

### Data analysis

The average iCAHE instrument scores (SD) for the three testers, and the domain and total AGREE II scores, were described for each guideline. Significant differences in the iCAHE scores (p < 0.05) between testers across the guidelines were determined using ANOVA models. It was not possible to determine the impact of tester experience on AGREE II domain or total scores because of the lack of a measure of variability (as per the scoring rubric) [15]. Construct validity was reported as Pearson’s correlations (95% Confidence Intervals) between percent of total scores for each guideline on each instrument, for pairs of testers. The average time (Standard Deviation) taken to score each guideline with each instrument was calculated per tester, and differences between testers and instruments were determined using ANOVA models. Microsoft Excel [20] and SAS [21] statistical software were used for these data analyses.

Given the small number of testers (3) and guidelines (6) used for this study, it was possible that unacceptable Type I and II errors has been incurred. A *post-hoc* power calculation was conducted using G-Power [22], based on an ANOVA repeat measures between factors model (α = 0.05, 18 guidelines, three testers, two instruments, 0.4 effect size, 0.7 correlation) to determine the degree of confidence which could be placed in the findings.

### Utility

Tester perspectives on using the guideline checklists were reported qualitatively.

## Results

### Guidelines tested

Details of the six randomly-selected clinical guidelines are provided in Table 1.

**Table 1:** Descriptions of guidelines used for psychometric testing

**Guideline reference**	**Purpose**
AANN and ARN (2011) Care of the Patient with Mild Traumatic Brain Injury: AANN and ARN Clinical Practice Guideline Series [23]*Country: USA*	This guideline was developed by the American Association of Neuroscience Nurses and the Association of Rehabilitation Nurses, and provides recommendations for nurses and institutions based on latest evidence for mild traumatic brain injury.
Barbosa (2012), Evaluation and management of mild traumatic brain injury: An eastern association for the surgery of trauma practice management guideline [24]*Country: USA*	This guideline updates an earlier 2001 edition. Recommendations for the management of mild traumatic brain injury are aimed at clinicians (primarily medical staff) working in acute care.
Brain Trauma Foundation (2012), Guidelines for the Acute Medical Management of Severe Traumatic Brain Injury in Infants, Children, and Adolescents-Second Edition [25]*Country: USA*	This guideline updates an earlier 2003 edition. Recommendations for the management of infants, children and adolescents with severe traumatic brain injury are aimed at acute care clinicians (primarily medical staff).
Golisz (2009), Occupational therapy practice guidelines for adults with traumatic brain injury [26]*Country: USA*	This guideline is aimed at occupational therapists. Recommendations are made for the evaluation, acute care and rehabilitation of adults with traumatic brain injury.
National Institute of Health & Clinical Excellence (NICE) (2007), Head injury: Triage, assessment, investigation and early management of head injury in infants, children and adults [27]*Country: UK*	This guideline is the update of an earlier 2003 edition. This guideline addresses assessment, investigation and early management of head injury. Separate advice is provided for adults and children (including infants).
Scottish Intercollegiate Guidelines Network (SIGN) (2013).Guidelines for traumatic brain injury rehabilitation [28]*Country: UK (Scotland)*	This guideline makes recommendations on the early management of patients with head injury, focusing on topics of importance throughout National Health Service, Scotland. Recommendations are made for the management of traumatic brain injury in adults and children

### Critical appraisal elements

The iCAHE instrument included questions that addressed four of the AGREE II domains (Scope and Purpose, Stakeholder Involvement, Rigour of Development and Clarity of Presentation). The iCAHE checklist deliberately did not address Applicability and Independence, as this was outside its remit to assess guideline construction quality. The AGREE II instrument did not include questions which addressed the new iCAHE instrument domains of Currency, Availability or Summary.

### Total quality scores

The percentage of total scores for each guideline from the iCAHE and AGREE II instruments are reported in Table 2. The six weighted domain scores for the AGREE II instrument are reported per guideline in Table 3. Higher percentage of total scores on both instruments, and higher domain scores on AGREE II, indicate better guideline quality. The AGREE II percentage of total score was substantially less than the iCAHE total percentage score for four of the six guidelines, although the scores were similar for SIGN [28] and Golisz [26]. Golisz [26] and AANN and ARN [23] had identical average iCAHE total scores, but differing scores for the AGREE II instrument. On closer inspection, the AANN and ARN [23] guideline had lower AGREE II scores for domains 5 and 6, than Golisz [26] (Applicability, and Editorial Independence, respectively). The questions in these domains are not represented in the iCAHE checklist (Table 4), which would perhaps account for the differences between instruments in the percentage of total possible scores reported in Table 2.

**Table 2:** **Average % total iCAHE scores (Standard Deviation) (over the three testers) for the six guidelines, and % total score considering all 23 questions in the AGREE II instrument, using the scoring rubric**[15]

**Guideline**	**iCAHE**	Rank*	**AGREE II**	**Rank**
	**Total % score**		**Total % score**^**§**^	
AANN and ARN [23]	73.8 (4.1)	4	*55.9*	6
Barbosa [24]	71.4 (0.0)	5	*56.7*	5
Brain Trauma Foundation [25]	92.9 (0.0)	3	78.1	3
Golisz [26]	*73.8 (14.9)*^+^	4	74.5	4
NICE [27]	97.6 (4.1)	2	84.9	2
SIGN [28]	100.0 (0.0)	1	97.3	1

*The guidelines are ranked by quality for each instrument.

^§^NB The % total AGREE II scores have no measure of variance.

^**+**^Significant differences between testers (p < 0.05) are noted in italics for the iCAHE instrument.

**Table 3:** Scaled domain scores (%)* derived from three testers, as per AGREE II scoring rubric[15]

	**Domain1**	**Domain2**	**Domain3**	**Domain4**	**Domain5**	**Domain6**
AANN and ARN Clinical Practice Guideline [23]	66.7	64.8	45.8	77.8	9.7	41.7
Barbosa (Eastern Association of the Surgery of Trauma) [24]	81.5	29.6	49.3	75.9	27.8	36.1
Brain Trauma Foundation [25]	92.6	57.4	89.6	92.6	26.4	80.6
NICE [27]	98.1	92.6	94.4	92.6	48.6	83.3
SIGN [28]	98.1	100.0	100.0	96.3	95.8	88.9
Golisz [26]	100.0	61.1	76.4	90.7	51.4	22.2

*NB The % domain scores have no measure of variance.

**Table 4:** Comparison of questions in AGREE II and iCAHE instruments relevant to domains

	**iCAHE**	**AGREE II**
***AGREE II Domain 1:*** Scope & Purpose	Q13 Are the purpose and target users of the guideline stated?	Q1. The overall objectives of the guideline are specifically described
		Q2. The health questions covered by the guideline are specifically described
		Q3. The population to whom the guideline is meant to apply is specifically described
		Q6. The target users are clearly defined
***AGREE II Domain 2:*** Stakeholder involvement	Q11. Are the developers clearly stated?	Q4. The guideline development group includes individuals from all relevant professional groups
	Q12. Does the qualifications and expertise of the guideline developers link with the purpose of the guideline and its end users?	Q5. The views and preferences of the target population have been sought
	Q7. Does the guideline provide an outline of the strategy used to find underlying evidence?	Q7. Systematic methods were used to search for the evidence
	Q8. Does the guideline use a hierarchy to rank the quality of the underlying evidence?	Q8. The criteria for selecting the evidence are clearly described
	Q9. Does the guideline appraise the quality of the evidence which underpins its recommendations?	Q9. The strengths and limitations of the body of evidence are clearly described
	Q10. Does the guideline link the hierarchy and quality of underlying evidence to each recommendation?	Q10. The methods for formulating the recommendations are clearly described
***AGREE II Domain 3:*** Rigour of Development		Q11. The health benefits, side effects and risks have been considered in formulating the recommendations
		Q12. There is an explicit link between the recommendations and the supporting evidence
		Q13. The guideline has been eternally reviewed by experts prior to its publication
		Q14. A procedure for updating the guideline is provided
***New iCAHE instrument Domain:*** Currency	Q4. Is there a date of completion available?	
	Q5. Does the guideline provide an anticipated review date?	
	Q6. Does the guideline provide dates for when literature was included?	
***AGREE II Domain 4:*** Clarity of Presentation	Q14. Is the guideline readable and easy to navigate?	Q15. The recommendations are specific and unambiguous
		Q16. The different options for management of the condition or health issues are clearly presented
		Q17. Key recommendations are easily identifiable
***AGREE II Domain 5:*** Applicability		Q18. The guideline describes facilitators and barriers to its application
		Q19. The guideline provides advice and/or tools on how the recommendations can be put into practice
		Q20. The potential resources implications of applying the recommendations have been considered
		Q21. The guideline presents monitoring and/or auditing criteria
***AGREE II Domain 6:*** Editorial Independence		Q22. The views of the funding body have not influenced the content of the guideline
		Q23. Competing interests of guideline development group members have been recorded and addressed
***New iCAHE instrument Domain*:** Availability	Q1. Is the guideline readily available in full text?	
	Q2. Does the guideline provide a complete reference list?	
***New iCAHE instrument Domain:*** Summary	Q3. Does the guideline provide a summary of its recommendations?	

### Ranking guidelines by quality

Considering the quality ranking of each guideline with each instrument, the SIGN guideline [28] was ranked best overall by both instruments, and in the AGREE II domains (ranking first in Domains 2–6 (Stakeholder Involvement, Rigour of Development, Clarity of Presentation, Applicability and Editorial Independence) and second in Domain 1 (Scope and Purpose)). The NICE guideline [27] was the second best overall ranked on both instruments, and it ranked 2 or 3 for all six AGREE II domains. In subsequent decreasing ranked order of total iCAHE and AGREE II scores, and the AGREE II domains, were the Brain Trauma Foundation guideline [25] and then Golitz [26]. The rankings of the remaining two guidelines (Barbosa [24], AANN and ARN [23]) were reversed in iCAHE and AGREE II, however they were both poorly ranked irrespective of which quality assessment instrument was used.

### Differences between tester iCAHE scores

There was total agreement between testers using the iCAHE instrument for three guidelines of different quality (SIGN [28] (high quality), Brain Trauma Foundation [25] (moderate quality) and Barbosa [24] (poorest quality). There was some disagreement between testers (SD 4.1) for two guidelines (NICE [27] (moderate - good quality), and AANN and ARN [23] (poorer quality). There was however, a significant difference between testers for one poorer quality guideline (Golisz [26], with the novice tester scoring significantly higher than the other testers.

### Prospective scoring bias

Apart from SIGN [28], no other guideline had sequentially similarly-scaled scores for subsequent domains (See Table 3). Thus a high score for Domain 1 (Scope and Purpose) was not an indication of overall guideline quality, and did not necessarily result in high scores for subsequent domains. This suggests that there was little or no sensitivity to initial high or low scoring by the testers. As an example, whilst most guidelines had high Domain 1 scores (Scope and Purpose), most had low scores for Domain 2 (Stakeholder Involvement) and Domain 5 (Applicability). Half the guidelines had low scores for Domain 6 (Editorial Independence), indicating that these areas require further attention by guideline developers. On the other hand, Domain 1 (Scope and Purpose), Domain 3 (Rigour of Development) and Domain 4 (Clarity of Presentation) generally scored highly, which suggests that the testers all considered that they well explained.

### Construct validity

There was a strong positive correlation between the percentage of total scores across six guidelines and three testers, when comparing the two instruments (r = 0.89, df = 4, p < 0.05) (critical r value = 0.812). This finding, and the consistency of quality rankings reported in Table 2 between the two instruments supports the construct validity of the iCAHE instrument in capturing the important items of guideline quality described in the AGREE II instrument. Considering percentage of total iCAHE scores for pairs of testers, there was a stronger positive correlation between Testers 1 and 2, than between either Tester 1 or 2, and Tester 3 (See Table 5). Less convincing correlations were evidenced by broader 95% CI round the Pearson r correlation coefficients.

**Table 5:** Paired-tester correlations between % total scores on the iCAHE instrument (Pearson r values, 95% CI)

**iCAHE checklist**	**Tester 1**	**Tester 2**	**Tester 3**
***Tester experience level***	***High***	***Moderate***	***Low***
Tester 1		0.97 (0.75 to 0.99)	*0.75 (-0.16 to 0.98)*
Tester 2			0.86 (0.16 to 0.98)
Tester 3			

NB non-significant correlations are indicated by italics.

### Making definitive judgements

**Figure 1:**
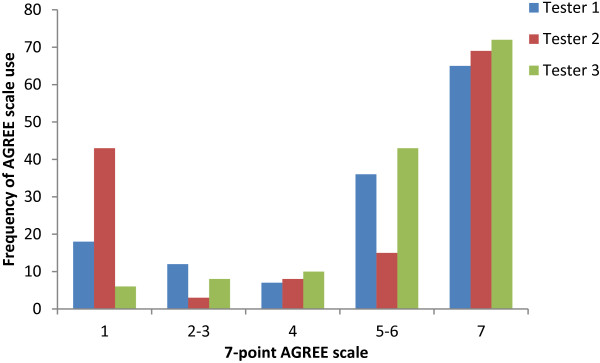
Frequency of use of AGREE II item 7-point scale, comparing the three testers’ aggregated raw scores for all six guidelines.

The iCAHE instrument requires a definitive response to each of 14 questions (either Yes or No). There is no other scoring option. On the other hand, the AGREE II scale is divided into low (1 = “strongly disagree”), 2–3, (4 = middle point “neither agree nor disagree”), 5–6 and the top end of the scale (7 = “strongly agree”). Thus AGREE II instrument provides two definitive options (No = 1 and Yes = 7) with ranked ‘disagree’ through to ‘agree’ options in between (2–6). Considering the definitive options (1 or 7) within the raw scores for the 23 AGREE II questions, for each guideline, there was considerable variability between testers, as outlined in Figure 1. Moreover, the least experienced researcher, Tester 3, did not use the bottom end of the scale (1–3) as much as the more experienced researchers did, particularly Tester 2. All three testers similarly used the top end of the scale (suggesting that either it is easier to make a judgement that a guideline complied with an item than not, or that the guidelines were generally compliant with the AGREE II questions). The moderately experienced researcher, Tester 2, seemed to dominate the ends of the scale more than Testers 1 and 3. This could perhaps be explained if it was assumed that Tester 1 understood the subtleties of the AGREE II criteria better than the other testers, and could sensitively score the quality of compliance with each item, whilst Tester 3 in her inexperience may have ‘hedged her bets’ and scored more towards the middle of the scale when a definitive 1 or 7 answer was not obvious.

Post-hoc power calculations indicated that this study of the psychometric properties of the new iCAHE instrument was under-powered (0.49) and should therefore be considered as a pilot. To be better powered (say at 0.8), future studies of the psychometric properties of the iCAHE instrument should include at least twice as many testers, and guidelines. Moreover, the guidelines chosen for future testing should include more poorer quality ones to ensure that the iCAHE instrument is sensitive across the spectrum of quality.

### Utility

All three raters indicated that the iCAHE tool was simpler and quicker to use than the AGREE II tool. Simplicity was identified in the number of iCAHE assessment questions (14) and the binary scoring options, compared with the 7-point scale for 23 questions in AGREE II. Whilst there were minimal within-tester differences in time taken to score with iCAHE, or AGREE II instruments, there were significant between-tester differences (p < 0.05) when using either instrument. Not surprisingly, the novice tester was the slowest on both instruments, taking on average, five minutes per guideline (range 3–7 minutes) with the iCAHE instrument, and up to 20 minutes per guideline using the AGREE II instrument (average 18 minutes (15–20 minutes range)). The most experienced tester consistently took 3–4 minutes per guideline using the iCAHE instrument, and 10–12 minutes per guideline using the AGREE II instrument. Tester 2 scores sat in between (4–6 minutes with iCAHE, 12–16 minutes with AGREE II). All testers indicated that they found the better quality guidelines easier and quicker to score than the poorer quality ones, because compliance with assessment items were more readily identifiable and reported in the better quality guidelines. However such ease of scoring was not reflected in the time taken. All testers indicated that much of the additional scoring time required for AGREE II instrument was spent in determining the appropriate score on the 7-point scale.

## Discussion

This paper reports promising psychometric properties of a new, clinically-focused clinical guidelines quality assessment instrument (the iCAHE Guideline Quality Scoring Instrument), compared with the research-focused AGREE II instrument. Despite the underpowered nature of this study, the findings from three testers with different experiences, assessing six clinical guidelines of moderate to good quality, suggest that the iCAHE critical appraisal instrument has the potential for good clinical utility and sound psychometric properties. It thus represents a viable critical appraisal approach for clinical guidelines for time-poor clinicians, policy-makers or managers.

To establish its psychometric properties and clinical utility, the iCAHE Guideline Quality Checklist was compared with the widely cited clinical guideline quality assessment instrument, AGREE II [15]. This instrument is not designed for, nor intended to be used by, clinicians, policy-makers or managers, as evidenced by the number of included questions, and its scoring system. There was however, correlation of question purpose between the two instruments in four domains (Scope & Purpose, Stakeholder involvement, Underlying evidence/Rigour, and Clarity). The iCAHE instrument included three additional domains (Currency, Availability, and Summary), and the only domains which were covered by the AGREE II instrument that were not addressed by the iCAHE instrument were Applicability and Editorial Independence. These had been purposely excluded from the iCAHE instrument during its development as being ‘next steps’ in contextualisation and implementation.

Six randomly-selected clinical guidelines in a similar diagnostic area (brain injury) [23-28] were used in this research. The quality of these guidelines ranged from moderate to good, and thus they may not have presented sufficient challenge in guideline quality assessment to establish the sensitivity of the iCAHE instrument across a range of guideline quality. Future research should not only include more guidelines and testers, but should include guidelines with poor quality, to ensure comprehensive opportunities to test the sensitivity of the iCAHE instrument.

Congruent with its application to busy clinical and policy environments, the 14 item iCAHE instrument uses a simple, binary form scoring system which can be readily summed and reported as a total raw score (or percentage) of 14. Time taken to score a clinical guideline approximates 3–5 minutes irrespective of the skill of the assessor. On the other hand, the AGREE II score requires value judgement using a 1–7 level scoring system, multiple assessors and the application of a scoring rubric to determine quality scores in six domains of 23 questions. Moreover, it is not recommended that a total AGREE II score is calculated, or raw scores used, although this was done for this paper to facilitate comparison between instruments. We believe that evidence supporting our claims of the clinical utility of the iCAHE instrument is provided in Figure 1, which outlines the difficulty that the novice guideline assessor had in making decisions about scoring in the AGREE II scale midpoints (3–5). Given this and the non-significant differences in iCAHE scores found between the three testers (moderate to excellent agreement for 17 of the 18 guideline assessments), it seems that the iCAHE instrument could be applied by anyone, with no prior experience or training. We also suggest that the iCAHE Guideline Quality Checklist may be simpler, more efficient and less prone to ‘guessing’ than the AGREE II instrument.

## Conclusion

The ultimate goal of evidence-based practice is to improve the quality and safety of health care. For this to occur, the current best evidence should be presented in a believable and readily implementable form for clinicians, policy-makers and managers. Clinical practice guidelines provide a useful mechanism to present current best evidence to clinical and policy end-users to ensure that their decision making is evidence-based. To be useful however, these guidelines need to be readily available and accessible, and their quality needs to be able to be efficiently assessed by busy end-users.

We propose that the iCAHE Guideline Quality Scoring Instrument provides a clinically-acceptable alternate to the AGREE II instrument to assess the quality of clinical guidelines, in clinical practice and policy settings. It can be completed relatively quickly by one individual, and it does not require specific training prior to use. It also does not pose the user with questions regarding ‘partial compliance’ with quality assessment items (as required in the AGREE II instrument) and it does not require a scoring rubric to produce an overall scaled score.

Once a guideline has been identified as having good methodological quality using the iCAHE Guideline Quality Scoring Instrument, end-users are encouraged to consider relevance, applicability and implementation issues. We believe that this is a simpler, staged approach for time-poor end-users in clinical environments than considering all these elements at the one time. Unless a clinical practice guideline has good methodological quality, there is little point in considering how to contextualise or implement it.

## Competing interest

The authors declare they have no competing interests.

## Authors’ contributions

KG, SM and SK conceptualised the instrument, and were involved in its initial construction process. KG, SM, OT, LL, ZM and KB subsequently tested the draft instrument in trial guideline critical appraisal processes in the iCAHE Clinical Guidelines Clearinghouse. ZM has subsequently scored all included guidelines on the iCAHE website with the checklist. KG, KB and JL were involved in identifying clinical guidelines used for the testing processes reported in this paper.KG, JD and EK undertook all independent scoring on the included guidelines, and EK undertook statistical analysis and reporting. The paper was drafted by KG and EK, and all authors contributed to subsequent writing and review. All authors approved version of the paper submitted to review, and all have approved the revised version.

## Pre-publication history

The pre-publication history for this paper can be accessed here:

http://www.biomedcentral.com/1471-2288/14/63/prepub

## Supplementary Material

Additional file 1 iCAHE Guideline Quality Check List. Click here for file
