# Supplementary material for: Efficient clinical evaluation of guideline quality: development and testing of a new tool
Source: BMC Med Res Methodol. 2014 May 10;14:63. doi: 10.1186/1471-2288-14-63 (PMC4033487; doi:10.1186/1471-2288-14-63)
Supplement: Additional file 1 — iCAHE Guideline Quality Check List. [file 1471-2288-14-63-S1.pdf]

iCAHE Guideline Quality Check List

Guideline:

Guideline producer:

Link:

Availability	Comments
Is the guideline readily available in full text?	(1)
Does the guideline provide a complete reference list?	(1)
Does the guideline provide a summary of its recommendations?	(1)
**Dates**	
Is there a date of completion available?	(1)
Does the guideline provide an anticipated review date	(1)
Does the guideline provide dates for when literature was included?	(1)
**Underlying Evidence**	
Does the guideline provide an outline of the strategy they used to find underlying evidence?	(1)
Does the guideline use a hierarchy to rank the quality of the underlying evidence?	(1)
Does the guideline appraise the quality of the evidence which underpins its recommendations?	(1)
Does the guideline link the hierarchy and quality of underlying evidence to each recommendation?	(1)
**Guideline developers**	
Are the developers of the guideline clearly stated?	(1)
Does the qualifications and expertise of the guideline developer(s) link with the purpose of the guideline and its end users?	(1)
**Guideline purpose and users**	
Are the purpose and target users of the guideline stated?	(1)
**Ease of use**	
Is the guideline readable and easy to navigate?	(1)
**Score**	TOTAL /14

International Centre for Allied Health Evidence

International Centre for Allied Health Evidence (iCAHE)
City East Campus, North Tce, Adelaide
University of South Australia
